# Radiotherapy resistance in chondrosarcoma cells; a possible correlation with alterations in cell cycle related genes

**DOI:** 10.1186/s13569-019-0119-0

**Published:** 2019-05-28

**Authors:** Yvonne de Jong, Martha Ingola, Inge H. Briaire-de Bruijn, Alwine B. Kruisselbrink, Sanne Venneker, Ieva Palubeckaite, Bram P. A. M. Heijs, Anne-Marie Cleton-Jansen, Rick L. M. Haas, Judith V. M. G. Bovée

**Affiliations:** 1 Department of Pathology, Leiden University Medical Center, Albinusdreef 2, 2333 ZA Leiden, The Netherlands; 2 Center for Proteomics and Metabolomics, Leiden University Medical Center, Leiden, The Netherlands; 3 Department of Radiation Oncology, Leiden University Medical Center, Leiden, The Netherlands; 4 Department of Radiation Oncology, The Netherlands Cancer Institute, Amsterdam, The Netherlands

**Keywords:** Chondrosarcoma, Radiotherapy resistance, Cell cycle alterations

## Abstract

**Background:**

Conventional chondrosarcomas are malignant cartilage tumors considered radioresistant. Nevertheless, retrospective series show a small but significant survival benefit for patients with locally advanced disease treated with radiotherapy. And, in daily practice when considered inoperable their irradiation is an accepted indication for proton beam radiotherapy. Therefore, we investigated the sensitivity of chondrosarcoma cell lines and -tissue samples towards radiotherapy and screened for biomarkers to identify predictors of radiosensitivity.

**Methods:**

Proliferation and clonogenic assays were performed in chondrosarcoma cell lines after γ-radiation in combination with mutant IDH1 inhibitor AGI-5198. In addition, glutathione levels were measured using mass spectrometry. Chondrosarcoma tumor explants were irradiated after which γ-H2AX foci were counted. Mutation analysis was performed using the Ion AmpliSeq™ Cancer Hotspot Panel and immunohistochemical staining’s were performed for P-S6, LC-3B, P53, Bcl-2, Bcl-xl and Survivin. Results were correlated with the number of γ-H2AX foci.

**Results:**

Chondrosarcoma cell lines were variably γ-radiation resistant. No difference in radiosensitivity, nor glutathione levels was observed after treatment with AGI-5198. Irradiated chondrosarcoma patient tissue presented a variable increase in γ-H2AX foci compared to non-radiated tissue. Samples were divided into two groups, high and low radioresistant, based on the amount of γ-H2AX foci. All four highly resistant tumors exhibited mutations in the pRb pathway, while none of the less radioresistant tumors showed mutations in these genes.

**Conclusions:**

Chondrosarcoma cell lines as well as primary tumors are variably radioresistant, particularly in case of a defective Rb pathway. Whether selection for radiotherapy can be based upon an intact Rb pathway should be further investigated.

**Electronic supplementary material:**

The online version of this article (10.1186/s13569-019-0119-0) contains supplementary material, which is available to authorized users.

## Background

Chondrosarcomas are a heterogeneous group of cartilage producing tumors of which the most prevalent subtype is conventional chondrosarcoma (85%). More rare subtypes include dedifferentiated chondrosarcoma (10%), mesenchymal chondrosarcoma (2%), clear cell chondrosarcoma (2%), and periosteal chondrosarcoma (1%). Conventional chondrosarcoma can be subdivided into three histological grades, representing the most important prognostic factor. Atypical cartilaginous tumors (previously referred to as grade I) have a relatively good prognosis, exhibiting a 10 years survival of 83%. Probability of surviving 10 years is about 64% for grade II chondrosarcomas and about 29% for grade III chondrosarcomas [1–3]. Based on their location conventional chondrosarcoma can be further subdivided into central (85%) and peripheral (15%) subtypes. Rare chondrosarcoma subtypes dedifferentiated, mesenchymal and clear cell chondrosarcoma occur also centrally in the bone [4–6], while periosteal chondrosarcoma is located and originates from the periosteal surface of the bone [3]. In this study, we focused on central chondrosarcomas, as these are of highest prevalence. Central chondrosarcomas carry mutations in the genes encoding the enzymes isocitrate dehydrogenase 1 or -2 (IDH1 or IDH2) [7–9] in approximately 50% of cases, resulting in production of the oncometabolite d-2-hydroxyglutarate (D-2HG).

The only curative treatment for patients with chondrosarcoma is radical surgery, since they are considered resistant towards conventional chemo- and radiotherapy. For this reason, linear accelerator based (conventional) radiotherapy is only given to patients with metastatic disease, incomplete resection or inoperable tumors in difficult sites like the base of skull or the sacrum [2, 10]. Several causes have been suggested for chondrosarcoma radioresistance, such as the relative hypoxic chondrosarcoma microenvironment impairing the induction of reactive oxygen species (ROS) and DNA damage, or their relatively slow dividing rate (though not applicable to high grade tumors). Additionally, knock down of anti-apoptotic Bcl-2 family members Bcl-2, Bcl-xl and XIAP has been shown to increase cell death after radiation in chondrosarcoma cell lines [11]. Furthermore, restoration of p16 activity sensitized chondrosarcoma cell lines to radiotherapy [12]. More recently, a role for *IDH* mutations has been found in predicting radiotherapy response in glioma due to altered redox responses in IDH mutant-compared to wild type cells thereby enhancing radiosensitivity [13].

A recent retrospective analysis suggested that a subgroup of chondrosarcoma patients with locally advanced, unresectable disease showed a favorable overall survival after conventional radiotherapy [10]. As chondrosarcoma patients are not commonly treated with radiotherapy, prognostic biomarkers for radiosensitivity were investigated using in vitro and ex vivo methods. Therefore, the aim of our study was to examine whether sensitivity to γ-radiation can be observed in central conventional chondrosarcoma cell lines by determining clonogenic survival and γ-H2AX foci induction after radiation. In addition, radiosensitivity of chondrosarcoma patient samples was determined by counting γ-H2AX foci after ex vivo radiation [14, 15]. Mutation and expression analyses were performed to investigate prognostic biomarkers for radiosensitivity which could then be used to select patients for radiotherapy.

## Methods

### Cell culture

Conventional chondrosarcoma cell lines JJ012 (Grade II, *IDH1* mutant) [16], SW1353 (Grade II, *IDH2* mutant) (ATCC) and CH2879 (Grade III, *IDH* wild type) [17] were cultured in RPMI-1640 (Gibco, Invitrogen Life-Technologies, Scotland, UK) supplemented with 10% Fetal Calf Serum, at 37 °C in a humidified incubator (5% CO_2_). Short tandem repeat analysis was performed before and after completion of experiments to confirm identity of the cell lines by using the Cell ID Gene Print 10 system (Promega Benelux BV, Leiden, The Netherlands). Mycoplasma tests were performed on a regular basis.

### Compounds

The specific mutant IDH1 inhibitor AGI-5198 (14624, Cayman Chemicals, Michigan, USA) was dissolved in DMSO according to the manufacturer’s instructions and stored in − 20 °C. AGI-5198 was used at a concentration of 10 µM since our group previously showed that this leads to a complete inhibition of D-2HG production [18]. (2R)-Octyl-α-hydroxyglutarate (16366, Cayman Chemicals), a cell-permeable derivative of D2-HG, was freshly dissolved in PBS before use and used at a final concentration of 250 µM.

### Clonogenic assays

Chondrosarcoma cell lines SW1353 and JJ012 were plated in optimal cell densities to obtain sufficient colonies after treatment. Cells were allowed to adhere overnight before treatment with a wide range (0, 1, 2, 4 or 6 Gy) of γ-radiation using a ^137^Cs source (YXLON, Comet technologies USA). Colony formation was assessed after 14 days of treatment by fixing and staining with 0.5% crystal violet/6% glutaraldehyde. Colonies were counted manually and the surviving fraction (SF) was calculated by normalizing towards the plating efficiency of untreated controls. The α/β ratios were calculated based on the linear quadratic model. α/β ratios describe the slope of the cell-survival curve; acute responding tissues show a higher ratio compared to late responding tissues.

### Viability assay

Chondrosarcoma cells were counted using a Burker Turk counting chamber and seeded in optimized cell densities in 96 well plates. After attachment overnight cells were irradiated with increasing doses. Seventy-two hours after radiation cell viability was measured using PrestoBlue viability reagent (Invitrogen, Life-Technologies, Scotland, UK) according to the manufacturer’s instructions. Fluorescence was measured at 590 nM using a Wallac plate reader (Victor3V, 1420 multilabel counter, Perkin Elmer, the Netherlands). Experiments were performed three times in triplicate.

### Cell proliferation measurement

To measure cell proliferation in real time the RTCA xCELLigence system (Roche Applied Sciences, Almere, the Netherlands) was used. JJ012, SW1353 and CH2879 cells were pre-treated for 72 h with either 10 µM AGI-5198 or 0.1% DMSO in T25 flasks. Thereafter, cells were plated in the presence of AGI-5198 or DMSO and left for 20 h to attach to the wells before low-dose (2 Gy) or high dose (4 Gy) γ-radiation. After 125 h the measurement was stopped, and the results were analyzed using RTCA software. Normalized cell index was calculated by normalizing against the amount of cells in each well after 20 h. Experiments were performed in duplicate and repeated at least two times.

### γ-H2AX staining of chondrosarcoma cell lines

Optimal cell amounts for JJ012 (550,000 cells), SW1353 (500,000 cells) and CH2879 (1,000,000 cells) cell lines were cultured on alcohol sterilized APES coated slides in a culture dish. IDH mutant chondrosarcoma cell lines SW1353 and JJ012 were pre-treated with 10 µM AGI-5198 or 0.1% DMSO for 72 h, in order to inhibit mutant activity, and IDH wild type chondrosarcoma cell line CH2879 was pre-treated with 250 µM D-2HG for 24 h, in order to mimic mutant activity. After overnight attachment cells were radiated to 5 Gy, in order to achieve highest response without significant damage to sample, and fixed after 2 or 24 h with buffered 4% formaldehyde (VWR Chemicals) at 37 °C. The procedure was performed as described previously [19]. Slides were stained with γ-H2AX antibody (JBW301, Millipore) for 60 min, washed, then a mixture of Alexa fluor 657 labelled secondary antibody and 0.5 µM Hoechst 33342 was applied for 60 min. Slides were covered with ProLongGold antifade reagent and were examined using a confocal microscope by taking tile scans of areas of interest using ZEN light software. Pictures were exported in TIF format and loaded into FoCo, a previously published format using Image J and Matlab software [20].

### Mass spectrometry measurements of glutathione

Chondrosarcoma cell lines JJ012, SW1353 and CH2879 were seeded in triplicate in T25 culture flasks and allowed to adhere overnight. *IDH* mutant chondrosarcoma cell lines JJ012 and SW1353 were pre-treated with 10 µM AGI-5198 for 72 h, while *IDH* wildtype cell line CH2879 was pre-treated with 250 µM D-2HG for 24 h. Samples were treated with 0 or 5 Gy, in order to correlate with γ-H2AX results. Cells were harvested by scraping after 1 h. For each individual replicate 1x10^5^ cells were fixed overnight using ice-cold acetone. The supernatant was then removed and dH_2_O added (10 µL), followed by a freeze–thaw cycle (10 s dry ice-cooled MeOH, 2 min 37 °C heating block). After 2 freeze thaw cycles glutathione was extracted with EtOH (50 µL) and the supernatant was mixed 1:1 (v:v) with matrix solution (*N*-(1-naphthyl) ethylenediamine dihydrochloride in 70% MeOH) for analysis. Samples were measured using a 9.4T SolariX XR Fourier transform ion cyclotron resonance (FT-ICR) mass spectrometer (Bruker Daltonics, Bremen, Germany) equipped with a CombiSource™ and a dynamically harmonized ParaCell™. The mass spectrometric analysis was performed in negative ion mode in the *m/*z-range 101.7–1000 Da, with a 1 M data point transient (0.3670 s duration) and an estimated resolving power of 66,000 at *m/z* 400. Spectra were acquired of 10 averaged scans, acquired in a random pattern covering the entire spot, where each scan consisted of 500 laser shots (laser power 70%, 1000 Hz frequency). Spectra were analyzed using DataAnalysis version 4.2 (Bruker Daltonics, Bremen, Germany). Using a custom algorithm, intensities of the compounds of interest were extracted and exported to Excel 2016 (Microsoft) for further analysis.

### Chondrosarcoma tissue samples

Chondrosarcoma patient samples (n = 9, see Tables 1 and 2) were obtained at surgery and collected in RPMI-1640 medium supplemented with 20% FBS and 1% P/S. All samples were coded according to the Dutch code of proper secondary use of human material as accorded by the Dutch society of pathology (Federa), and as approved by the LUMC ethical board (B17.019). Samples were processed by manual dissection into approximately 2 mm fragments and incubated on a rotation shaker at 37 °C in a humidified incubator (5% CO_2_). The tissue was treated to 5 Gy, consistent with the cell line treatment, and incubated for 2 or 24 h on a rotation shaker at 37 °C in a humidified incubator as described previously [21]. After overnight fixation with formalin, tissue was processed, embedded in paraffin and sections were cut and stained for γ-H2AXfoci.

**Table 1:** Patient samples treated with 5 gy of radiation

Sample	Grade	IDH mutation status	Other alterations	Mean + SEM basic foci level	Mean + SEM after 5gy radiation	Difference between means	P value
L5213	1	WT		3.053 ± 0.6277 n = 38	14.28 ± 1.737 n = 32	11.23 ± 1.733	< 0.0001
L5678	3	IDH1 R132C		0.2295 ± 0.07165 n = 61	8.239 ± 1.477 n = 46	8.01 ± 1.284	< 0.0001
L5676	2	IDH2 R172S		3.625 ± 0.9297 n = 32	9 ± 1.493 n = 34	5.375 ± 1.784	0.003712
L5541	Dediff	IDH1 R132S		2.378 ± 0.5747 n = 90	6.702 ± 1.225 n = 57	4.324 ± 1.212	0.0005
L5546	2	WT	CDKN2A deletion	3.982 ± 0.9036 n = 56	6.846 ± 1.172 n = 52	2.864 ± 1.468	0.0537
L5302	3	WT	CDKN2A deletion	2.246 ± 0.4531 n = 61	5.082 ± 0.7412 n = 73	2.836 ± 0.9108	0.0023

Foci were counted 2 h after treatment

**Table 2:** Patient samples treated with 5 gy of radiation

Sample	Grade	IDH mutation status	Other alterations	Mean + SEM basic foci level	Mean + SEM after 5gy radiation	Difference between means	P value
L5847	2	WT		2.644 ± 0.7399 n = 59	7.467 ± 1.526 n = 30	4.823 ± 1.501	0.0018
L5718	3	IDH1 R132L	CDKN2A deletion	2.437 ± 0.584 n = 87	4.088 ± 0.6256 n = 102	1.651 ± 0.8659	0.0580
L5850	2	WT	RB1 deletion	0.6327 ± 0.1409 n = 98	1.55 ± 0.1809 n = 140	0.9173 ± 0.2463	0.0002

Foci were counted 24 h after treatment

### γ-H2AX staining and quantification of paraffin embedded chondrosarcoma tissue

Sections were deparaffinized and antigen retrieval was performed using DAKO antigen retrieval solution pH 9.0 by boiling the slides in a microwave for 12 min. After cooling, slides were washed with TBS and blocking was performed using TBS/5% normal goat serum/1% BSA and 0.2% Triton-x-100. Anti γ-H2AX antibody (Millipore 2310355) was applied in a 1:1000 dilution and incubated overnight at 4 °C. Slides were washed with TBS and incubated with secondary labelled GAM Alexa fluor 647 antibody. Mounting was performed with ProLongGold containing DAPI (Thermofisher Scientific, Waltham, Massachusetts, USA). Positive control slides consisted of JJ012 cells radiated at 4 Gy as these displayed a consistent, high γ-H2AX signal. The cells were embedded in paraffin with the use of Shandon™Cytoblock™ Cell Block Preparation system (Thermofisher Scientific). Slides were analyzed using a confocal microscope by taking tile scans of areas of interest using ZEN light software. Pictures were exported in TIF format and loaded into FoCo, a previously published format using Image J and Matlab software [20]. The settings to quantify the foci were first optimized using the positive control slides, after which the complete series was analysed using the same settings.

### Immunohistochemical staining

Protein expression of Bcl-2, Bcl-xl, Survivin, P-S6, P53 and LC-3B were evaluated in irradiated control patient samples (see Additional file 1: Table S1). Immunohistochemistry was performed according to standard laboratory methods as previously described [22]. All slides were scored by two independent observers (JVMGB, YDJ) using a scoring system assessing staining intensity (0 = no, 1 = weak, 2 = moderate, 3 = strong) as well as percentage of staining (0 = no, 1 = 1–24%, 2 = 25–49%, 3 = 50–74%, 4 = 75–100%) [23]. This scoring system was used for all the different proteins.

### Mutation analysis

Frozen tissue of chondrosarcoma patients was collected at surgery and DNA was isolated using the wizard genomic DNA purification kit (Promega, Madison, WI, USA) according to the manufacturer’s instructions. Samples were subjected to next generation sequencing using the in-house developed Ion AmpliSeq™ Cancer Hotspot Panel v2 (Life Technologies, Thermo Fisher Scientific, USA, catalog number 4475346) as described previously [24, 25]. The following genes and regions were analysed on an Ion Torrent PGM/Proton for all samples (exons between brackets): *ARAF* (7,10); *CTNNB1* (1,2,4,7,8,12,15); *KRAS* (2–4); *NRAS* (2–4); *HRAS* (2–3); *BRAF* (11,15); *EGFR* (3,7,15,18–21); *GNAQ* (5); *GNAS* (8–9); *H3F3A* (2); *H3F3B* (2); *IDH1* (4); *IDH2* (4); *KIT* (2,9-18); *MYD88* (3b,5), *MUTYH* (7,13); *PDGFRA* (12,14,15,18,23); *PIK3CA* (2,5,6–10,14,18,21); *POLE* (9,11,13,14); *RET* (10–12,15,16); *TP53* (1–11). In addition hotspot regions in the following genes were also included: *ABL1; AKT1; ALK; APC; ATM; CARD11; CD79A; CD79B; CDH1; CDKN2A; CSF1R; CTNNB1; ERBB2; ERBB4; EZH2; FBXW7; FGFR1; FGFR2; FGFR3; FLT3; GNA11; HNF1A; JAK2; JAK3; KDR; MET; MLH1; MPL; NOTCH1; NPM1; PTEN; PTPN11; RB1; SMAD4; SMARCB1; SMO; SRC; STK11; VHL*. Libraries were prepared with 10 ng of genomic DNA, and each sample was uniquely barcoded. Ion Proton chips were prepared using the Ion Chef System. The unaligned bam files generated by the Proton sequencer were mapped against the human reference genome (GRCh37/hg19) using the TMAP 5.0.7 software with default parameters (https://github.com/iontorrent/TS). The Torrent Variant Caller (TVC)-5.0.2 was used for variant calling and variant interpretation was done using Geneticist Assistant (http://softgenetics.com/GeneticistAssistant_2.php) as described. Chromosomal gains and deletions were assessed by calculating the median base coverage per amplicon, which was normalized using the median value of all amplicons in that sample.

## Results

### Chondrosarcoma cell lines are variably resistant to γ-radiation

**Fig. 1:**
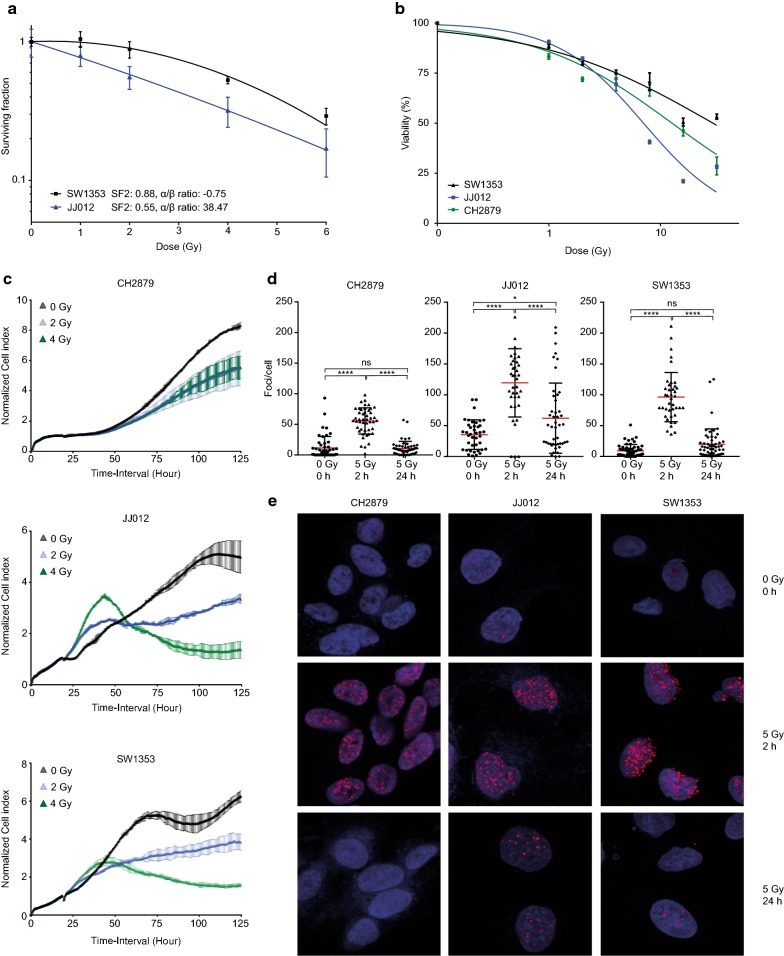
Chondrosarcoma cell lines exhibit variable γ-radioresistance. **a** Colony forming assay of SW1353 and JJ012 chondrosarcoma cells. **b** Viability of JJ012, SW1353 and CH2879 cells, 72 h after treatment with increasing doses of γ-radiation measured using presto blue viability reagent. **c** Normalized cell index of CH2879, JJ012 and SW1353 cells irradiated to 0 (black), 2 (blue) or 4 Gy (green) radiation. **d**, **e** γH2AX foci/cell in CH2879, JJ012 and SW1353 cells 2 and 24 h after 5 Gy radiation.****P values < 0.0001, *P values < 0.05

Clonogenic assay SF2 values (surviving fraction of cells after 2 Gy radiation) suggested that JJ012 cells (SF2 0.55) were more radiosensitive compared to SW1353 (SF2 0.88) (Fig. 1a). JJ012 cells showed a high α/β ratio of 38.47, in contrast to SW1353 with a very low α/β ratio of − 0.75, suggesting that SW1353 cells may benefit from hypofractionation. CH2879 cells were unable to form colonies and were therefore not included in this assay. In addition, dose response curves were made to assess viability after 72 h. JJ012 cells were most sensitive followed by the CH2879 and SW1353 cells (Fig. 1b). An X-CELLigence assay was performed to determine the effect on proliferation in real time after 2 or 4 Gy of radiation. All cell lines showed a reduction in proliferation after radiation. However, CH2879 cells showed a similar response when irradiated to 2 or 4 Gy, while JJ012 and SW1353 showed a dose response relationship (Fig. 1c). The amount of double strand breaks was determined by quantifying γ-H2AX foci 2 and 24 h after radiation treatment. JJ012, SW1353 and CH2879 cells exhibited an increase in foci after 2 h, which was decreased again after 24 h in SW1353 and CH2879 cells (Fig. 1d–e). Conversely JJ012 cells still showed a substantial significant amount of foci compared to non-radiated cells, indicating this cell line is less proficient in repairing double strand breaks and more sensitive to radiation.

### γ-Radiosensitivity of chondrosarcoma cell lines is not correlated with *IDH* mutation status

Inhibition of D-2HG production by JJ012 cells using the mutant IDH1 inhibitor AGI-5198 did not show any difference in radiosensitivity (Additional file 1: Figure S1A). In addition no differences were observed in proliferation capacity and foci formation between cells treated with AGI-5198 and radiation or cells treated with radiation only (Additional file 1: Figure S1B, C). Since previous reports [13] suggest that *IDH* mutant cells have a reduced capability of producing GSH, GSH levels were measured 1 h after radiation in combination with AGI-5198, for the mutant cell lines or D-2HG (oncometabolite resulting from an IDH mutation) for the wild type cell line. The most radioresistant SW1353 cells displayed the highest baseline levels of GSH, followed by CH2879 and JJ012, however no differences were observed between different treatment conditions, indicating that GSH levels are not influenced by D-2HG inhibition (Additional file 1: Figure S1D). These results suggest that IDH and D-2HG do not play a role in chondrosarcoma radiosensitivity.

### Chondrosarcoma samples that are more resistant towards radiotherapy have an increased incidence of mutations in cell cycle regulators

**Fig. 2:**
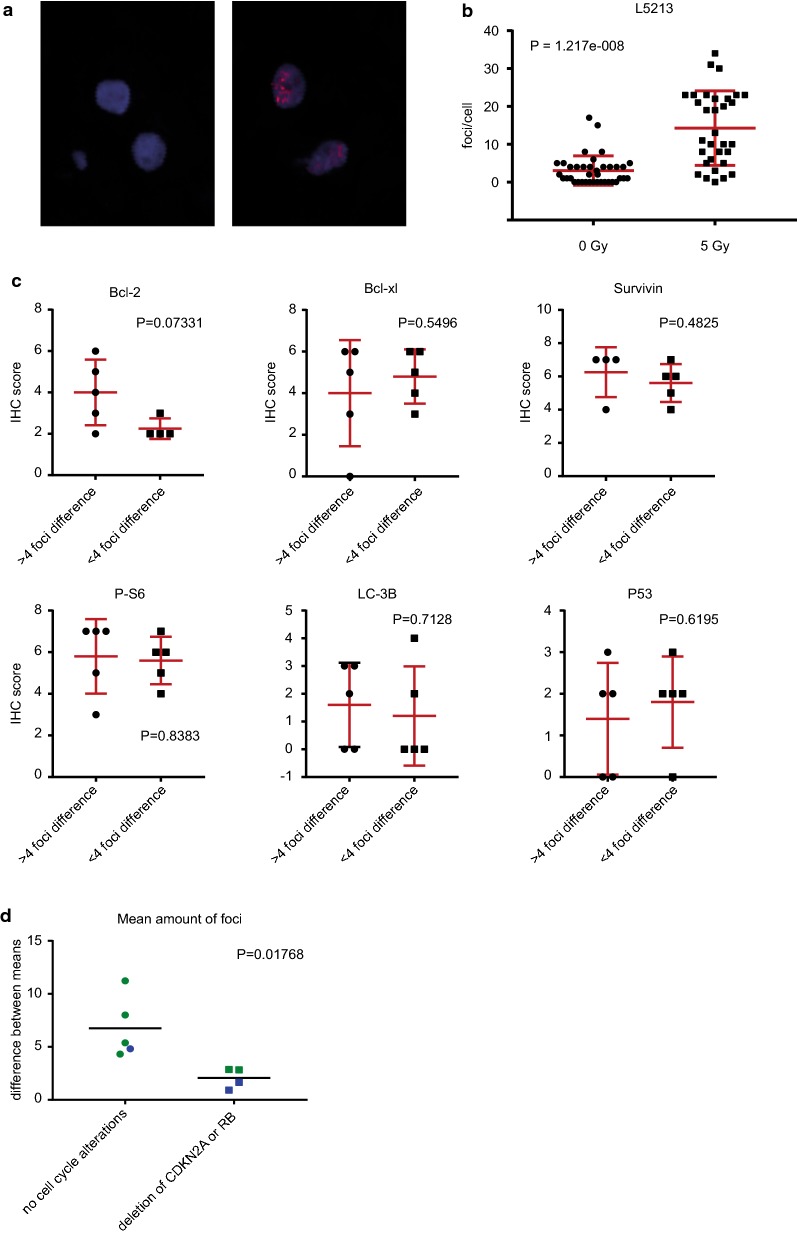
γ-Radioresistance in chondrosarcoma tissues correlates with mutations in cell cycle related genes. **a** L5213 chondrosarcoma tissue sample showing a large induction of γH2AX foci after5 Gy(right) compared to controls (right). **b** Amount of foci/cell in sample L5213 after 5 Gy radiation compared to controls. **c** Protein expression of Bcl-2, Bcl-xl, Survivin, P-S6, LC3B and P53 in highly radioresistant (< 4 foci difference) compared to less radioresistant (> 4 foci difference) chondrosarcomas. **d** Difference between mean amount of γH2AX foci before and after radiation in chondrosarcomas with and without alterations in *RB1* or *CDKN2A*. Each dot represents one sample. Samples in green indicate 2 h, while samples in blue indicate samples 24 h after radiation

Chondrosarcoma patient samples showed an increase in γ-H2AX foci after 5 Gy radiation treatment. In Tables 1, 2 and Additional file 1: Figure S2 quantified results are shown for nine chondrosarcoma explant tissue samples analyzed 2 or 24 h after treatment with 5 gy of radiation. Radiation response was heterogeneous across the samples; the largest difference between control and radiated samples was 11.2 foci (sample L5213, Fig. 2a, b), while the smallest difference was 0.9 foci. Samples analyzed 24 h after radiation treatment in general showed a lower amount of foci compared to samples analyzed 2 h after radiation treatment. Samples that showed a difference of 4 or more foci were subjected to the less radioresistant group, while samples that showed < 4 foci difference were designated as radioresistant for further analysis. This cutoff value was taken for both the 2 h and 24 h groups and both time points were analyzed together since sample size impeded separate analyses. The division between more or less radioresistant was made prior to mutation analysis. No correlation was observed between histological grade or *IDH* mutation status and the amount of γ-H2AX foci, consistent with the results obtained in the chondrosarcoma cell lines (Table 1). Mutation analysis was performed on 50 known cancer related genes and expression of Bcl-2, Bcl-xl, Survivin, P-S6, LC3B and P53, previously identified to play a role in chondrosarcoma [26–30], was determined using immunohistochemistry. No significant difference was observed in protein expression of selected markers (Fig. 2c, Additional file 1: Figure S3), however three *CDKN2A* deletions (3/4) and one *RB1* deletion (1/4) were found in the highly radioresistant group and none in the less radioresistant group indicating that a defective Rb pathway may be able to impair the response to γ-radiation in chondrosarcoma (Fig. 2d, Tables 1 and 2). Interestingly a mutation in *CDKN2A* was also identified in the SW1353 cell line (Additional file 1: Table S2), which is the most radioresistant cell line.

## Discussion

Chondrosarcomas are relatively radioresistant tumors and therefore, after multidisciplinary discussions, very few of these patients are offered radiotherapy [10]. In this study we investigated the sensitivity of chondrosarcoma cells and tumor explants to γ-radiation. In addition, we screened for biomarkers that could select patients that might benefit from γ-radiation. Chondrosarcoma cell lines showed a heterogeneous response to radiotherapy with relatively high SF2 values [31]. JJ012 cells presented a SF2 value of 0.55, while SW1353 were more resistant showing a SF2 value of 0.88. Compared to cell lines of other tumor types, these values are relatively high, confirming chondrosarcoma radio resistance. A previous published study by Hamdi et al. showed a SF2 value of 0.64 for the SW1353 cell line [32]. This difference might be attributed to the fact that different methods were used to perform the clonogenic assay. In our study the cells were seeded prior to radiation, while in the study of Hamdi et al. the cells were subconfluently seeded in culture flasks and subsequently radiated and seeded. In addition the differences in culture conditions (normoxic vs hypoxic) can very well influence how cells respond to radiation treatment. Quantification of γ-H2AX foci showed that SW1353 and CH2879 cells were able to repair double strand breaks within 24 h after radiation while JJ012 cells were less capable of doing so, in line with the lower SF2 values observed in JJ012 cells.

In contrast to published studies in glioma [13, 33], we did not observe a correlation between radiosensitivity and *IDH* mutation status in chondrosarcoma. Inhibition of mutant IDH1 using AGI-5198 did not lead to any changes in radiosensitivity, nor in altered GSH levels. Also, no difference was observed in γ-H2AX foci formation between *IDH* mutant and *IDH* wild type chondrosarcoma explants. This indicates that, unlike the observations in gliomas, *IDH1* or *IDH2* mutations in chondrosarcoma do not correlate with radiosensitivity. This is in line with our previous results in which we also did not find any correlation between *IDH1* or -*2 *mutation status in sensitivity for inhibitors of glutaminolysis [34], NAD synthesis [35] or Bcl-2 family members in chondrosarcoma cells, while in other tumor types there was a clear difference in sensitivity [36–38]. This difference indicates that *IDH* mutations may have a tissue specific effect rather than a more general effect in different tumor types.

Our results suggest that deletions in cell cycle regulators *CDKN2A* and *RB1 *are associated with increased radioresistance in chondrosarcoma explant tissue. In line with this, the most resistant SW1353 cells harbored a mutation in the splice site region of *CDKN2A *in addition to mutations in *TP5*3 and a *kRAS* mutation [39]. *CDKN2A* is a gene encoding p16(INK4A) and p14(ARF), which are two tumor suppressor proteins controlling the cell cycle. P16 inhibits CDK4 and CDK6, two inhibitors of Rb1 phosphorylation, while p14-ARF protects p53 from being broken down by inhibiting MDM2. Alterations in the pRB pathway have been described in the majority of high grade conventional chondrosarcomas [40–43]. Previous studies in chondrosarcoma cell lines (CS-7, CS-8, CS-9) showed that restoring P16 expression, and thereby increasing Rb1 phosphorylation, resulted in an increased radiosensitivity [12], in line with our results. Although we see a clear difference in the amount of foci in tumors with and without deletions in *CDKN2A* or *RB1*, this study is based on a small heterogenous group of chondrosarcomas and measurements are taken after 2 or 24 h. In addition the threshold of 4 foci/cell is taken arbitrarily. This complicates making firm conclusions based on this data and further studies should focus on; including more patients and analyze amount of foci after 24 h to determine the damage remaining after DNA repair. In addition when radiotherapy is included in the treatment plan, follow the response towards radiation and correlate this towards mutation status.

In contrast to our findings in chondrosarcoma, deletion of *RB1* has been described to enhance radiosensitivity in breast, prostate and bladder cancer [44–47]. This observation might therefore be tissue and context specific; only a limited number of cancer types have been investigated. In addition, pRb has multiple functions, not only in the cell cycle but also in chromatin organization, transcription patterns, metabolic pathways and the proteome [48]. Several studies found that loss of pRB expression resulted in an increase in glutamine consumption and an increased glutamine incorporation into GSH. Upon RB knock down cells increased the expression of glutamine transporters and upregulated glutaminase activity, indicating that the pRB pathway can regulate glutamine metabolism and that cells with inactivated pRB are potentially more sensitive for targeted anti-glutamine treatment [49–51]. Interestingly, we recently reported that interfering with glutamine metabolism can be a therapeutic target for high grade chondrosarcoma [34]. The SW1353 chondrosarcoma cell line was particularly sensitive for inhibition of glutaminase, which is in line with the high basal GSH levels measured in this cell line. We can hypothesize that the mutation in *CDKN2A* observed in this cell line might contribute to the glutamine dependence, however more research is needed to further investigate this.

In addition to conventional radiotherapy, research has been focusing increasingly on proton [^1^H] and carbon [^12^C] therapy, which have several advantages compared to γ-radiation. Both these beam qualities have dose distribution advantages, causing less damage to surrounding healthy tissues, making it possible to deliver higher doses to the tumor. Chondrosarcomas of the skull base and spine are increasingly treated with proton beam radiation, showing promising results [52]. Whether chondrosarcoma radiosensitivity differs between photon (γ-) beams and proton beams is subject for further investigation.

## Conclusion

In conclusion, this study confirms a heterogeneous radiosensitivity of chondrosarcoma cell lines and fresh explant tissue. In addition, we identified alterations in *CDKN2A/RB1* as predictors of decreased double strand break formation after radiotherapy in chondrosarcoma tissue, and although further research is necessary in a larger group, this suggests that these patients might benefit less from radiation therapy.

## Additional file

**Additional file 1.** Supplementary material.

## Data Availability

All data generated or analysed during this study are included in this published article (and its additional files).

## Abbreviations

D-2HG: d-2-hydroxyglutarate
IDH1 or IDH2: isocitrate dehydrogenase 1 or -2
ROS: reactive oxygen species
SF: surviving fraction
