# Supplementary material for: Radiotherapy resistance in chondrosarcoma cells; a possible correlation with alterations in cell cycle related genes
Source: Clin Sarcoma Res. 2019 May 28;9:9. doi: 10.1186/s13569-019-0119-0 (PMC6540537; doi:10.1186/s13569-019-0119-0)
Supplement: Supplementary file 1 — Additional file 1. Supplementary material. [file 13569_2019_119_MOESM1_ESM.pdf]

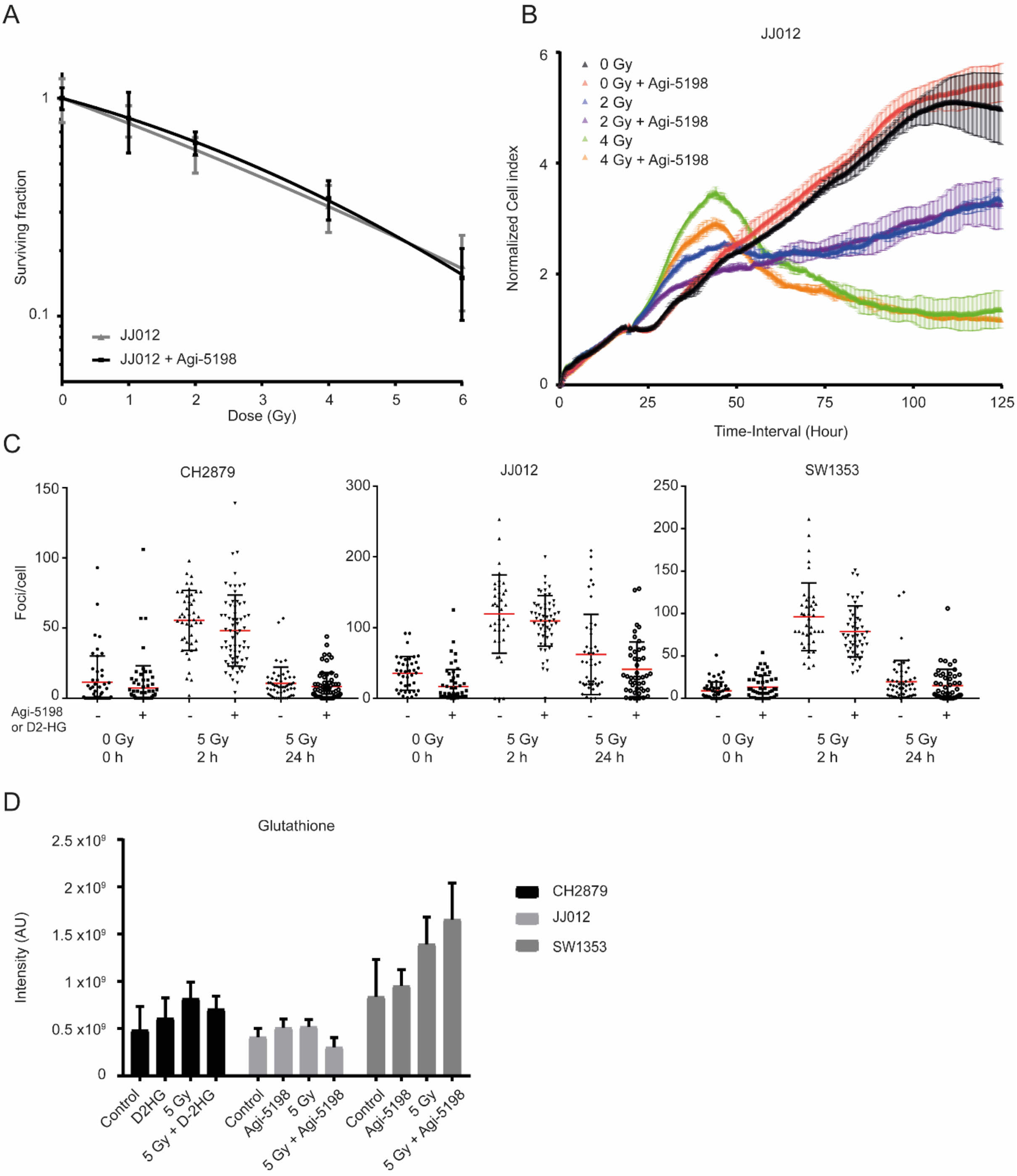

Additional file 1: Figure S1 1. No relation between *IDH* mutation and radiosensitivity in chondrosarcoma cell lines A) clonogenic assay of JJ012 cells with or without 10 µM mutant IDH1 inhibitor AGI-5198.**B)** normalized cell index of JJ012 cells treated with 0, 2 or 4 Gy radiation in combination with 10 µM mutant IDH1 inhibitor AGI-5198 or DMSO as a control. Black indicates 0 Gy, Red 0 Gy + AGI-5198, blue 2 Gy, Purple 2 Gy + AGI-5198, green 4 Gy, Orange 4 Gy + AGI-5198. **C)**

Amount of yH2ax foci in CH2879, JJ012 and SW1353 after 2 and 24h of treatment with 5 Gy radiation with and without 10 µM AGI-5198 or 250 µM D-2HG **D)** glutathione levels of chondrosarcoma cells treated with 10 µM AGI-5198 or 250 µM D-2HG measured 1h after radiation.

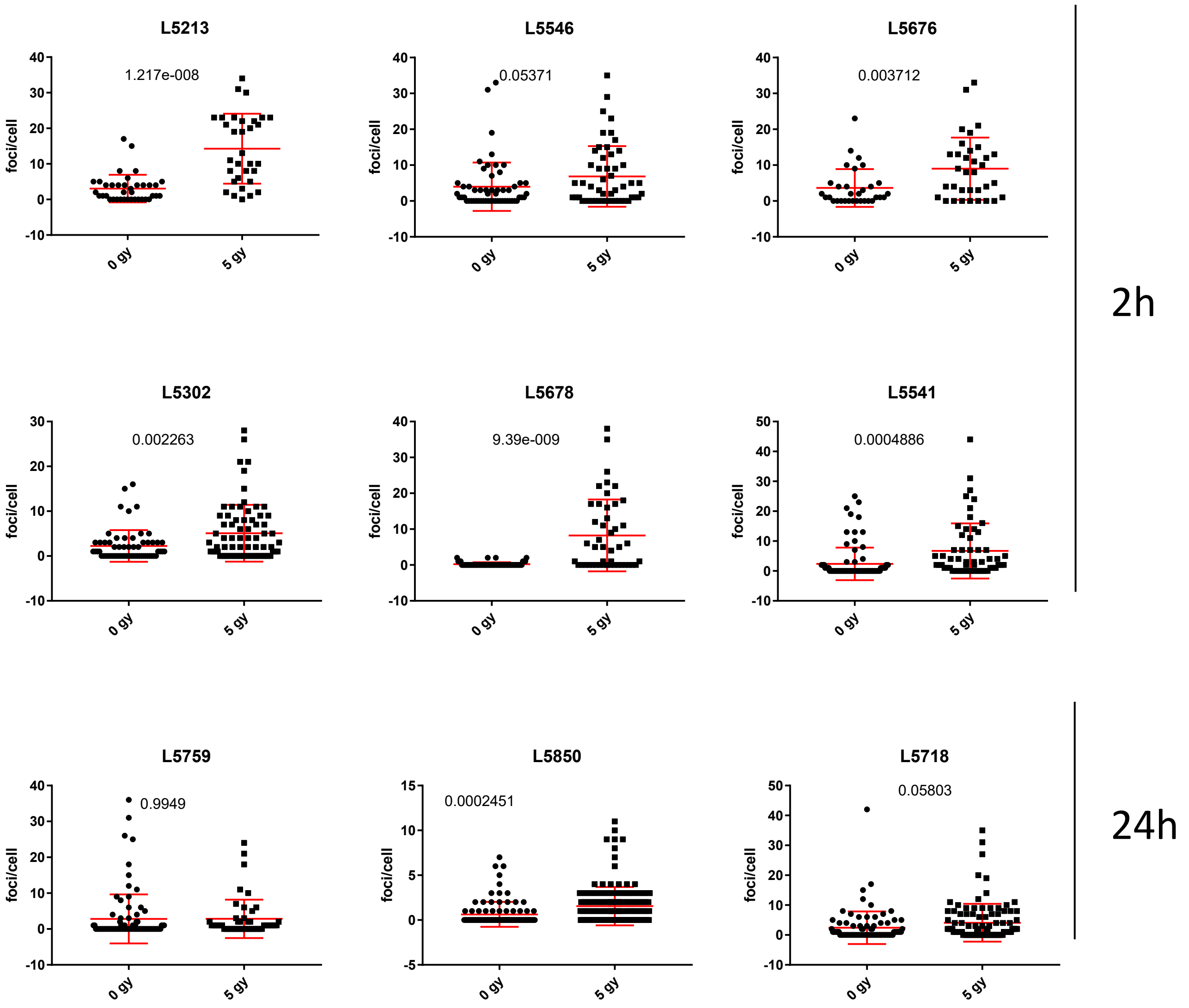

Additional file 1: Figure S2. γ-H2ax staining quantification of chondrosarcoma patient samples treated with 5 gy radiation. Significance was assessed by performing a T-test

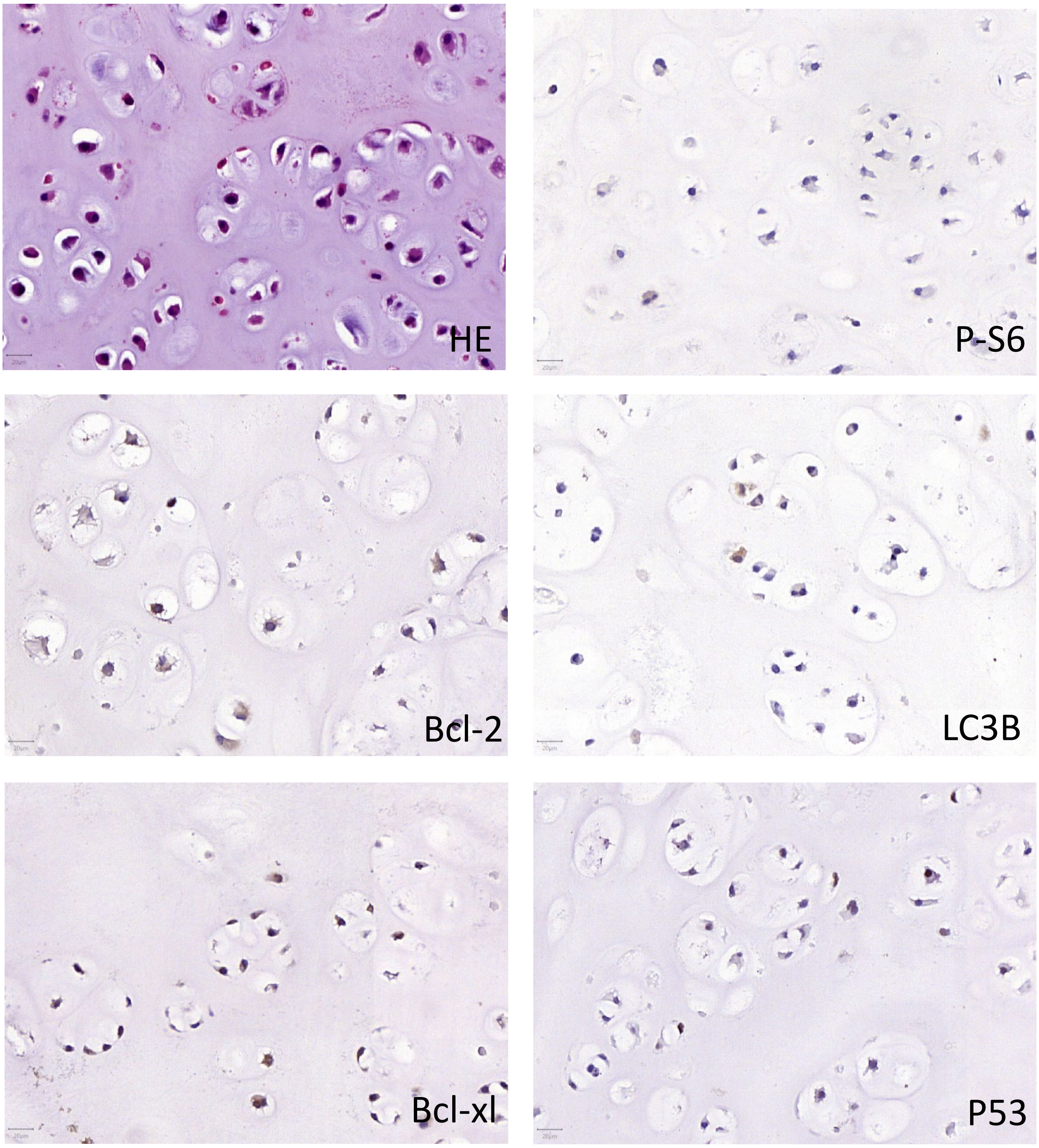

Additional file 1: Figure S3A L5847

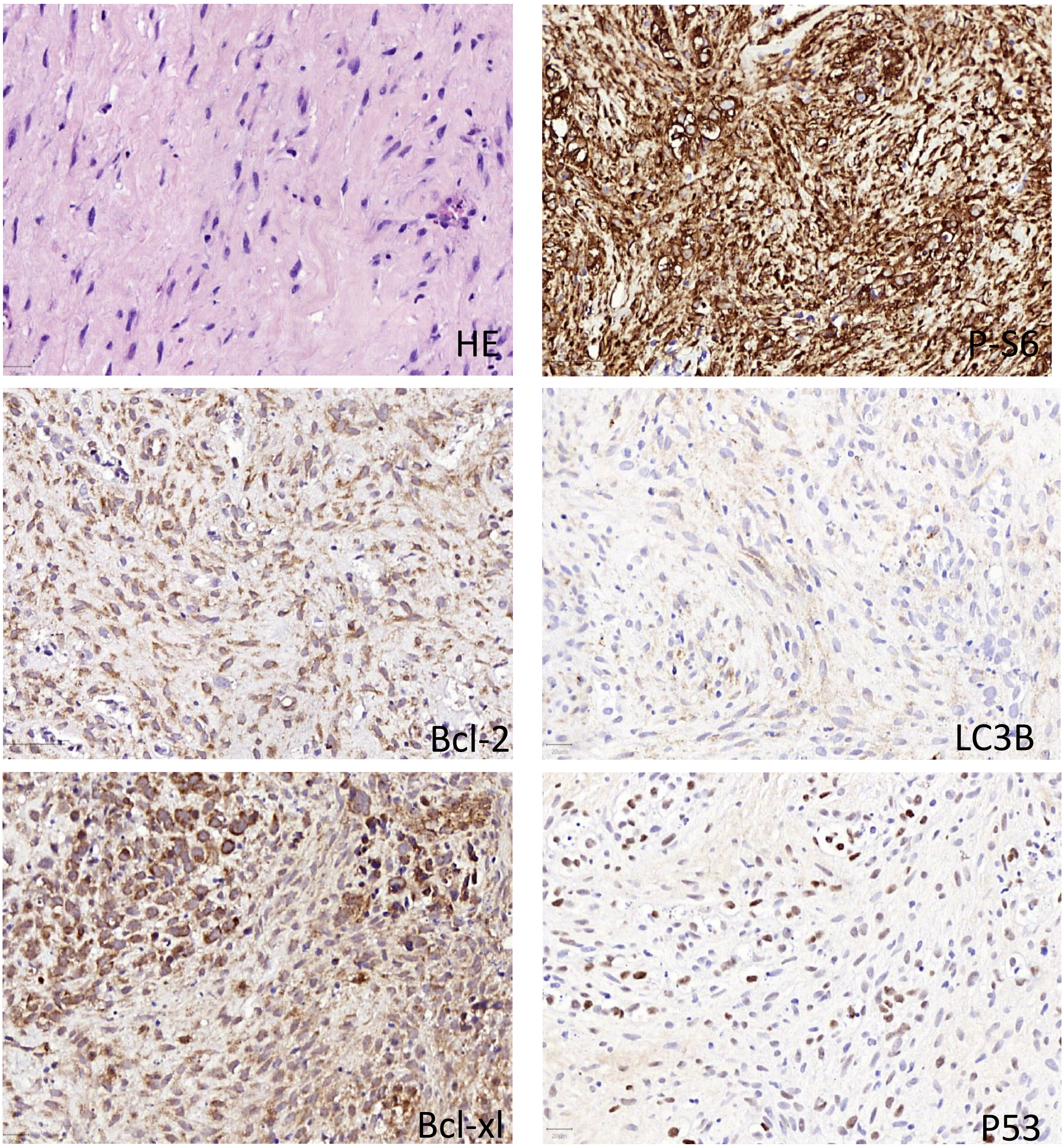

Additional file 1: Figure S3B L5541

Additional file 1: Table S1. Conditions of Immunohistochemical staining on chondrosarcoma tissue

Protein	Dilution	Blocking	Antigen Retrieval	Positive control	Manufacturer and clone
Bcl-2	1:100	-	Tris-EDTA	Tonsil	Dako clone 124
Bcl-xl	1:400	5% Milk	Citrate	Prostate	Cell signalling clone 54H6
Survivin	1:100	-	Citrate	Placenta	Cell signalling clone 71G4B7
P-S6	1:800	-	Citrate	Melanoma	Cell signalling clone D57.2.2E
P53	1:800	-	Citrate	Tonsil	Dako clone DO-7
LC3B	1:40000	5% Milk	Citrate	Cerebral cortex	Cell signalling Clone D11

Additional file 1: Table S2. Mutation analysis of chondrosarcoma cell lines. Results on *IDH* and *TP53* mutation status were published previously (de Jong et al. 2016).

	IDH1	*IDH2*	*TP53*	*CDKN2A*	*KRAS*	*PTEN*
SW1353	-	R172S	V203L	9:21971209 A-C*	G12C	-
JJ012	R132G	-	G199V	-	-	-
CH2879	-	-	S366A R273C#	-	-	R233Ter

# only found in 17% of cells

* mutation found in splice site of intron
